# Cladribine Use in Relapsing Multiple Sclerosis After 8–10 Years: Two Case Reports of Patients From the ORACLE-MS Study

**DOI:** 10.7759/cureus.74671

**Published:** 2024-11-28

**Authors:** Miguel Angel Hernandez, Rossana Abreu Rodriguez, Yessica Contreras Martin

**Affiliations:** 1 Neurology, Multiple Sclerosis Unit, University Hospital Nuestra Señora de la Candelaria, Santa Cruz de Tenerife, ESP

**Keywords:** case report, cladribine, immune reconstitution therapy, long-term effectiveness, multiple sclerosis

## Abstract

Cladribine is an immune reconstitution therapy for multiple sclerosis (MS) that selectively produces long-term reductions in highly pathological memory B cells, with temporary reductions in other B- and T-cell subsets, thereby restoring immune function close to baseline levels in the short term. Here, we describe two cases of relapsing MS (RMS) treated with a second course of cladribine. Both patients were initially diagnosed with clinically isolated syndrome and later enrolled in the ORACLE-MS and CLASSIC-MS studies. After receiving cladribine in ORACLE-MS, neither patient required additional treatment until RMS was diagnosed 8-10 years later. In each case, a second course of cladribine was administered, and both patients remained without clinical or radiological disease activity as per their most recent assessments (November 2023 and August 2023, respectively). No serious or unexpected adverse events were reported during follow-up. These cases provide further real-world evidence of the long-term effectiveness and safety of cladribine after a second course of treatment in patients with RMS.

## Introduction

Multiple sclerosis (MS) is a chronic inflammatory disease of the central nervous system (CNS), characterized by demyelinating lesions with axonal loss as the disease progresses [1] as well as various physical and cognitive symptoms [2]. Cladribine is an immune reconstitution therapy that selectively and transiently reduces CD19+ B- and T-cell levels, leading to the reconstitution of adaptive immune function [3]. In patients with MS, cladribine tablets (10 mg) are given as a cumulative oral dose of 3.5 mg/kg over two years (i.e., 1.75 mg/kg/year, separated by 12 months) [4]. Each treatment course comprises two weeks per year (five days of treatment weekly), one at the beginning of the first month and another at the beginning of the second month. If medically necessary (such as for recovery of lymphocytes), the treatment course in year 2 can be delayed for up to six months. Typically, after two years of cladribine therapy, no further treatment is required in years 3 and 4 [5].

The ORACLE-MS study (NCT00725985) investigated the effect of cladribine on conversion from clinically isolated syndrome (CIS) to clinically definite MS in adults [6]. In these patients, cladribine significantly delayed the conversion to clinically definite MS compared to placebo. A post-hoc analysis of the ORACLE-MS trial assessed the effect of cladribine in patients with a first clinical demyelinating attack who met newer diagnostic criteria (i.e., McDonald 2017) [7,8]. Compared with placebo, cladribine 3.5 mg/kg significantly reduced the time to relapse or three-month confirmed expanded disability status scale (EDSS) worsening in patients with early relapsing MS (RMS) [7]. In October 2011, the ORACLE-MS study was terminated prematurely, at which time all cladribine administration was discontinued due to issues with the production of cladribine tablets. Following termination, all patients completed a 24-week safety follow-up with a final end-of-trial visit [6].

The long-term efficacy of cladribine tablets beyond four years has been previously described in several clinical trials and real-world studies. In the Italian CLARINET-MS registry, >50% of patients with MS who had received cladribine in clinical trials were relapse-free five years after their last dose [9]. In the CLARITY and CLARITY-extension studies, cladribine showed benefits for up to six years, considering the gap between both studies in which no treatment was administered was a median duration of 40.3 weeks (range: 0.1-118) [10,11]. The phase IV CLASSIC-MS study (NCT03961204) prospectively evaluated the long-term efficacy, safety, and durability outcomes of cladribine treatment in patients who were enrolled in the parent studies (i.e., ORACLE-MS and CLARITY/CLARITY-EXT) [12]. An analysis of the CLASSIC-MS study found that during a 10.9-year follow-up period under routine clinical practice, 55.8% of patients who were treated with at least one dose of cladribine in the parent clinical trials did not require further disease-modifying therapies (DMTs) [12]. However, evidence of retreatment with cladribine in RMS is scarce [13].

Here, we present two case reports summarizing the clinical evolution of two patients (who presented consecutively) after receiving cladribine for early-stage MS in the ORACLE-MS study in 2010. Both patients subsequently entered the CLASSIC-MS study and experienced disease stability for 8-10 years until MS was retreated with a second course of cladribine, the outcomes of which are described here. A concise timeline of diagnosis, tests, and treatment is provided for Case 1 (nine years and 10 months between cladribine treatments) and Case 2 (eight years and two months between cladribine treatments) in the next section. The first demyelinating event occurred in July 2010 for Case 1 (monitoring continued until November 2023) and June 2010 for Case 2 (monitoring continued until August 2023). The duration of MS symptoms was 11 years and five months for Case 1 and eight years and one month for Case 2.

## Case presentation

Case 1

A 45-year-old female, who had smoked approximately 12 cigarettes per day for 15 years before presentation and had a history of six pregnancies, three spontaneous abortions, and no drug use, presented to our facility in July 2010 with loss of strength and sensitivity in the right hemibody (Table 1).

**Table 1:** Timeline of diagnoses, tests, and treatment in Case 1 CIS: Clinically isolated syndrome; IM: Intramuscular injection; IVC: Intravenous contrast; MRI: Magnetic resonance imaging; MS: Multiple sclerosis; RMS: Relapsing multiple sclerosis; SE: Weighted-spin echo; T2-FLAIR: T2-weighted-fluid-attenuated inversion recovery.

Date	Type of event/scan/treatment	Results
06 July 2010	1^st^ demyelinating event	Loss of strength and sensitivity of the right hemibody
08 July 2010	Lumbar puncture performed	Positive for oligoclonal bands
14 July 2010	Standard cranial MRI study performed with usual multiplanar sequences, diffusion sequence, and after IVC in multiplanar projections included	High signal areas of the T2 sequence, predominantly a paraventricular lesion in the left atrium with involvement of the splenium of the adjacent corpus callosum; target morphology was a central area of higher signal intensity in T2 of approximately 12 x 8 mm, with a peripheral area of lower signal intensity in T2, high diffusion (without apparent coefficient restriction) and annular enhancement, discrete, and predominantly peripheral after IVC. Other high signal intensity T2 left atrium lesions were found adjacent to the splenium and corpus callosum, paraventricular to the left frontal horn and in the semioval center, paraventricular to the right ventricular body. Diagnosis: CIS (no treatment administered).
13 October 2010	Enrolled in ORACLE-MS	Received course 1 of cladribine; cumulative dose: 5.25 mg/kg (high)
13 September 2011	ORACLE-MS terminated	Completed end-of-trial visit
May 2012	2^nd^ demyelinating event	Sensory-motor relapse of the left hemibody. Diagnosis: MS (treatment: dexamethasone 40 mg/day IM for 3 days)
June 2012	Follow-up visit	Symptomatic recovery
June 2014	Cranial MRI	No disease activity
October 2019	Non-enhanced cranial MRI	Two new lesions detected
July 2020	3^rd^ demyelinating event	Sensory-motor relapse of the left hemibody. Diagnosis: RMS (treatment: methylprednisolone 500 mg/day for 5 days)
26–30 August 2020	Enrolled in CLASSIC-MS Year 1, month 1	Started course 2 of cladribine 7 × 10-mg tablets (i.e., 70 mg total); 2–2–1–1–1 per day
26–30 September 2020	Year 1, month 2	7 × 10-mg tablets (i.e., 70 mg total); 2–2–1–1–1 per day
17 December 2020	Cranial MRI	No disease activity
15–19 October 2021	Year 2, month 1	7 × 10-mg tablets (i.e., 70 mg total); 2–2–1–1–1 per day
15–19 November 2021	Year 2, month 2	7 × 10-mg tablets (i.e., 70 mg total); 2–2–1–1–1 per day
2 December 2021	Cranial MRI with MS control protocol sequences after IVC acquired in axial T2-FLAIR, T2-SE, and T1-SE	New areas of signal alteration; one lesion in the left frontal corona radiata. No lesions after contrast. No cerebral atrophy. Diagnosis: RMS with activity data.
August 2022	Follow-up cranial MRI	No disease activity
November 2023	Cranial MRI	No disease activity

**Figure 1:**
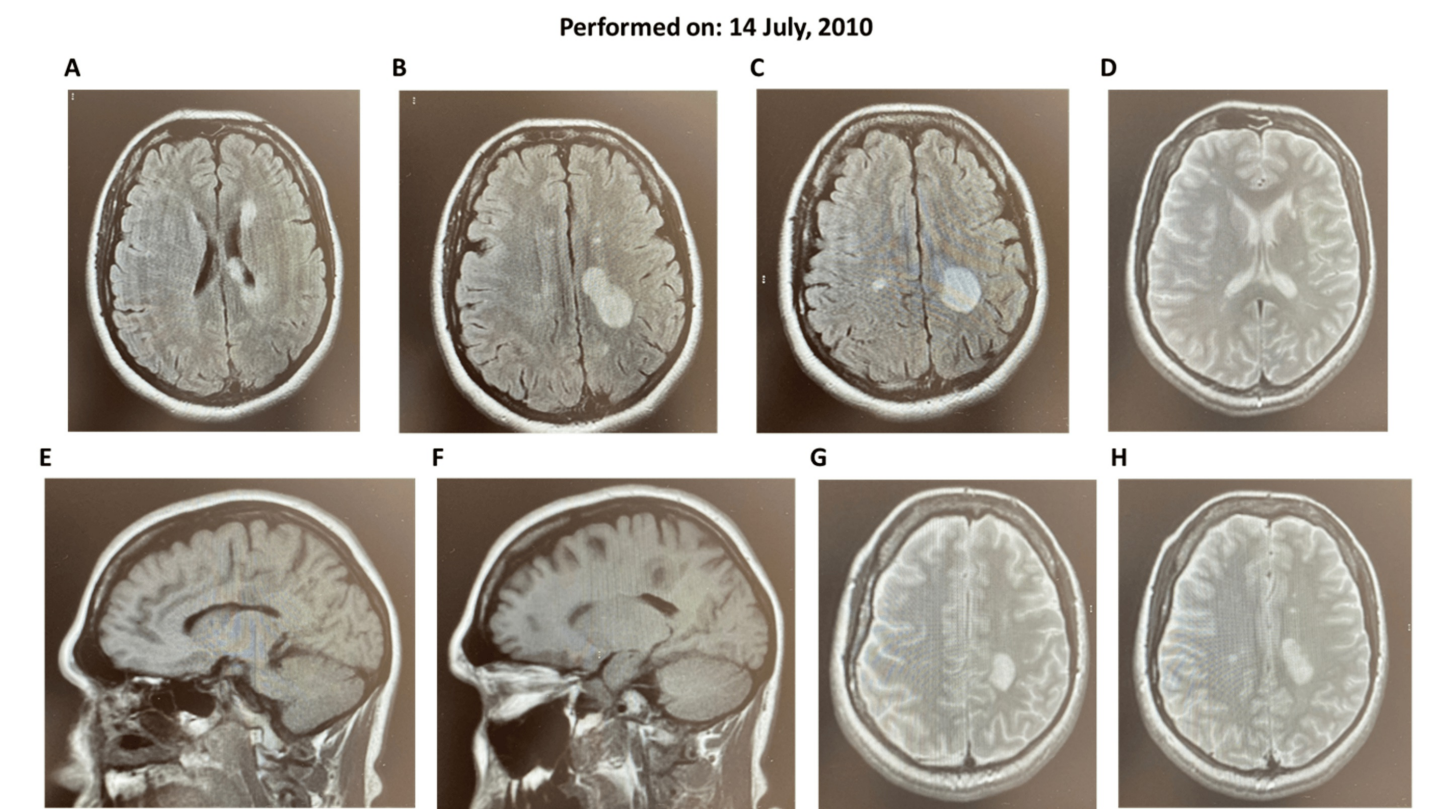
(A-H) Initial cranial magnetic resonance imaging scans for Case 1

Analytical studies and her autoimmunity profile were normal, but a lumbar puncture was positive for oligoclonal bands. A cranial MRI scan taken in July 2010 (Figure 1) revealed multiple lesions, predominantly a paraventricular lesion in the left atrium.

**Figure 2:**
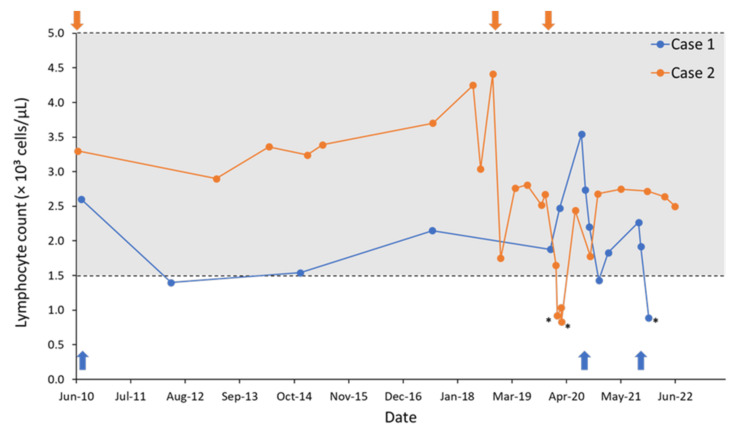
Lymphocyte count over time from the diagnosis of clinically isolated syndrome. Arrows indicate the cladribine administrations (months 1 and 2) for each patient; asterisks (*) denote grade 1 lymphopenia; and the gray area represents the normal range for lymphocyte count (1.5–5.0 × 10³ cells/μL).

Based on these findings, which suggested inflammatory processes and demyelination, she received a diagnosis of CIS. At this time, she had one gadolinium‐enhancing (Gd+) basal cell lesion and a lymphocyte count of 2.6 × 10^3^/μL (Figure 2).

**Figure 3:**
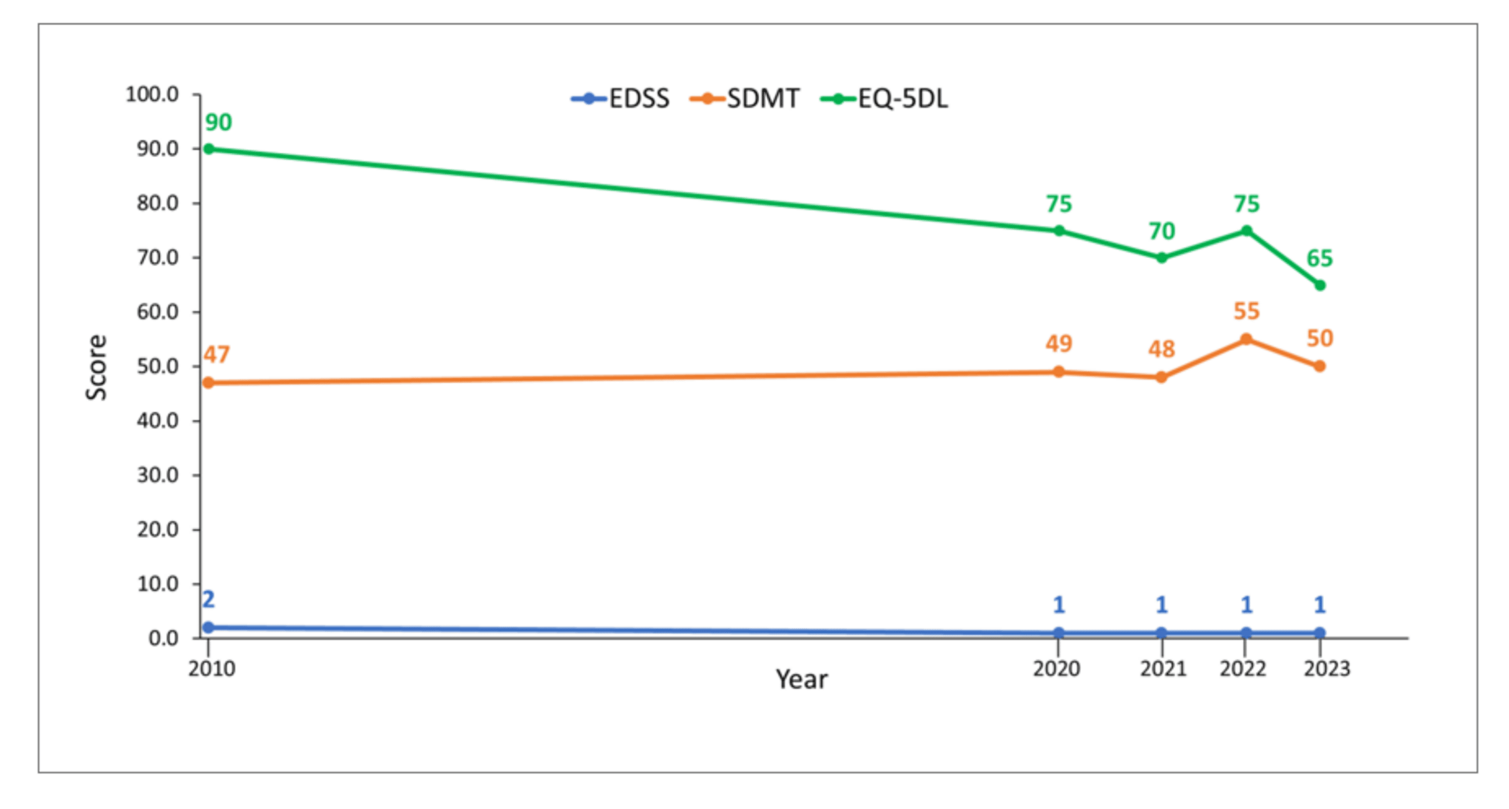
Expanded disability status scale (EDSS), EuroQol 5-dimension questionnaire (EQ-5DL), and symbol digit modalities test (SDMT) scores for Case 1 at different time points during the course of follow-up. EDSS scores are measured on a scale of 1–10, with higher scores indicating greater disability. The EQ-5DL is scored from 0 to 100, with lower scores reflecting more disability. The SDMT is scored from 0 to 100, with lower scores reflecting more disability.

She was then enrolled in the ORACLE-MS study in October 2010. Her EDSS score was 2, her EuroQol 5-dimension questionnaire (EQ-5DL) score was 90, and her Symbol Digit Modalities Test (SDMT) score was 47 (Figure 3).

At ORACLE-MS enrolment, the patient had not received any previous treatment for CIS as this was her first diagnosis of CIS. In accordance with the ORACLE-MS study protocol, she initially received cladribine 5.25 mg/kg (cumulative dose); the first dose was received on October 13, 2010. Cladribine was very well tolerated with no adverse events (AEs). Due to the premature termination of the ORACLE-MS study, the patient completed her end-of-trial visit on September 13, 2011.

In May 2012, she experienced a second demyelinating event, with a sensory-motor relapse of the left hemibody. She was diagnosed with MS and treated with dexamethasone for three days, after which she reported symptomatic recovery in June 2012. No disease activity occurred until October 2019, when two new lesions were discovered on a non-enhanced MRI scan, and RMS was diagnosed. A third demyelinating event (MRI scan identified a sensory-motor relapse of the left hemibody) in July 2020 was treated with a pulse of oral methylprednisolone. The patient received steroids because she did not wish to receive any MS-modifying treatment, as she wanted to become pregnant at the time.

**Figure 4:**
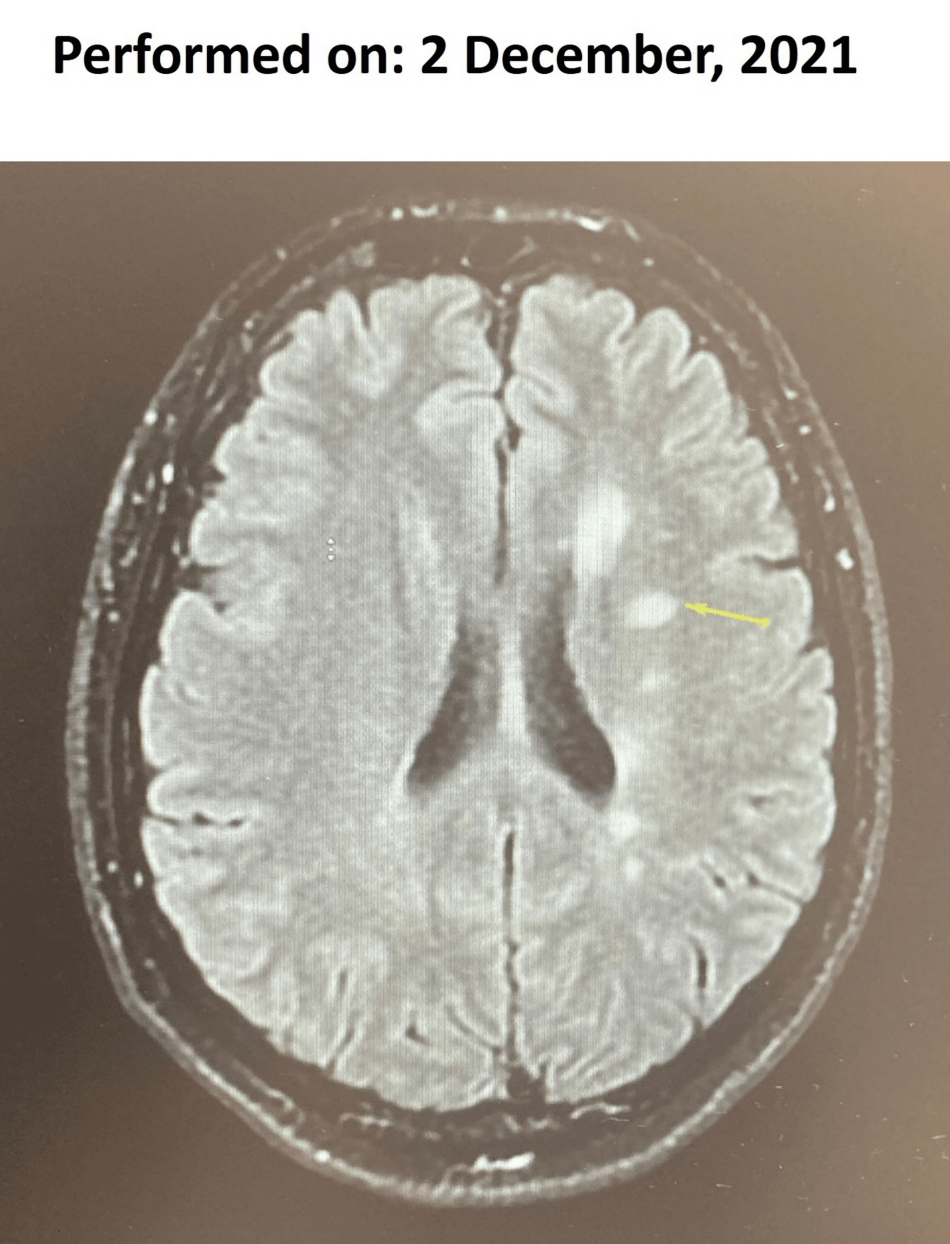
Subsequent cranial magnetic resonance imaging scan with contrast for Case 1

Nevertheless, a second course of cladribine to treat RMS was started in August 2020, and disease activity did not resume. Although this course of cladribine treatment was well tolerated, her EQ-5DL score and EDSS score had worsened by September 2021, and RMS activity resumed in December 2021 (Figure 4). However, in August 2022, almost nine months after the cladribine administration period had ended, a follow-up MRI showed no disease activity. As of August 2023, the patient had remained free of relapses and clinical progression; her EQ-5DL score had decreased to 65, and SDMT and EDSS scores showed little modification (Figure 3).

Changes in the patient’s lymphocyte count since her CIS diagnosis throughout the ORACLE-MS study and subsequent follow-up (11 years) are shown in Figure 2. Her lymphocyte count remained within the normal range until December 3, 2021, when she presented with grade 1 lymphopenia (lymphocyte count 0.89 × 10^3^/μL); no follow-up lymphocyte counts were performed, and the AE did not require any active management. No relevant changes were observed in lymphocyte levels until the last evaluation in November 2023.

Case 2

A 50-year-old man presented to our facility after experiencing his first clinical event (paresthesia in the lower limbs of both legs with discrete weakness) on June 9, 2010 (Table 2).

**Table 2:** Timeline of diagnoses, tests, and treatment in Case 2 CIS: Clinically isolated syndrome; Gd+: Gadolinium-enhanced; IVC: Intravenous contrast; MRI: Magnetic resonance imaging; MS: Multiple sclerosis; RMS: Relapsing multiple sclerosis.

Date	Type of event/scan/treatment	Results
9 June 2010	1^st^ demyelinating event	Paresthesia in the lower limbs of both legs with discrete weakness
18 June 2010	Cervicodorsal MRI performed	No demyelinating inflammatory lesions were detected; cranial MRI was recommended.
21 June 2010	Standard cranial MRI study performed with the usual multiplanar sequences, and diffusion sequences after IVC in multiplanar projections were included.	Multiple high-signal areas in T2 sequences, affecting infratentorial periventricular white matter (middle cerebellar and right cerebral peduncles) and subcortical areas, with morphological criteria of spatial dissemination indicative of MS. After IVC, at least two areas of predominantly annular enhancement were observed in the right cerebellar peduncle and homogeneous right parasagittal subcortical areas. Diagnosis: CIS (no treatment administered).
3 August 2010	Enrolled in ORACLE-MS study	Received course 1 of cladribine, cumulative dose: 3.50 mg/kg (low).
7 July 2011	ORACLE-MS study terminated	Completed end-of-trial visit
January 2015	2^nd^ demyelinating event, cranial MRI performed	Non-enhancing injury; lower limb paresthesia without weakness. Diagnosis: MS (no treatment administered)
August 2017	Follow-up visit	No disease activity
9 July 2018	Cranial MRI performed	One Gd+ right paraventricular lesion was detected. Diagnosis: RMS.
18–22 October 2018	Enrolled in CLASSIC-MS Year 1, month 1	Started course 2 of cladribine 7 × 10-mg tablets (i.e., 70 mg total); 2–2–1–1–1 per day
16–20 November 2018	Year 1, month 2	7 × 10-mg tablets (i.e., 70 mg total); 2–2–1–1–1 per day
September 2019	Cranial MRI	No disease activity
5–9 November 2019	Year 2, month 1	7 × 10-mg tablets (i.e., 70 mg total); 2–2–1–1–1 per day
5–9 December 2019	Year 2, month 2	7 × 10-mg tablets (i.e., 70 mg total); 2–2–1–1–1 per day
November 2020	Follow-up cranial MRI	No disease activity
June 2021	Follow-up cranial MRI	No disease activity
June 2022	Follow-up cranial MRI	No disease activity
August 2023	Follow-up cranial MRI	No disease activity

**Figure 5:**
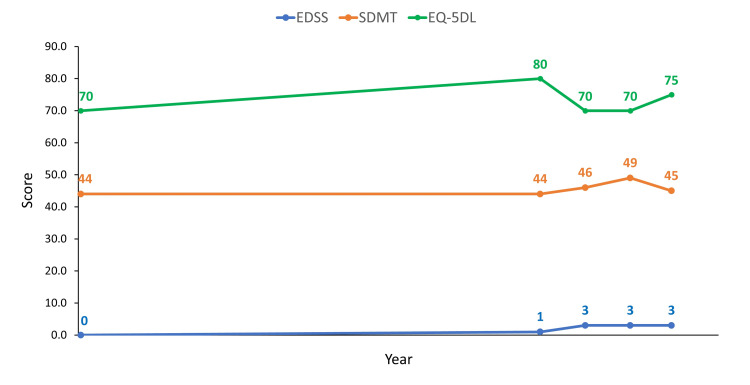
Expanded disability status scale (EDSS), EuroQol 5-dimension questionnaire (EQ-5DL), and symbol digit modalities test (SDMT) scores for Case 2 at different time points during the course of follow-up. EDSS scores are measured on a scale of 1–10, with higher scores indicating greater disability. The EQ-5DL is scored from 0 to 100, with lower scores reflecting more disability. The SDMT is scored from 0 to 100, with lower scores reflecting more disability.

At this time, he had an EDSS score of 0, an EQ-5DL score of 70, and an SMDT score of 44 (Figure 5).

**Figure 6:**
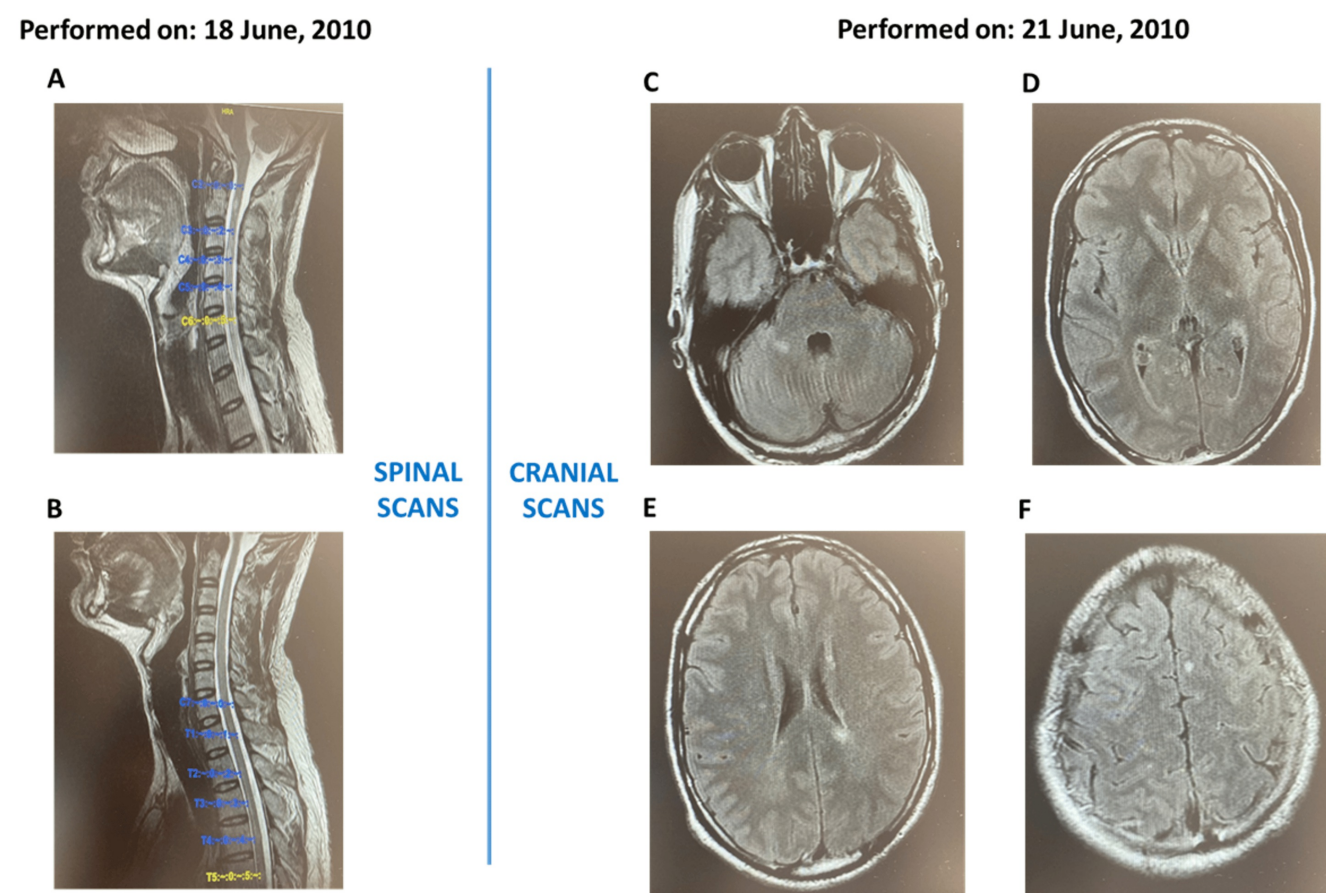
(A-F) Initial magnetic resonance imaging scans for Case 2

On June 18, 2010, a spinal MRI showed no inflammatory demyelinating diseases (Figure 6, Panels A and B). A cranial MRI scan that was performed with and without intravenous contrast revealed two Gd+ lesions, and CIS was diagnosed (Figure 6, Panels C-F).

The patient was a former smoker (10 years before the study entry) and had not received any previous treatment for CIS. He had no relevant family history, surgical interventions, or history of drug use. He was subsequently enrolled in ORACLE-MS, and according to the study protocol, he received a cumulative dose of cladribine 3.50 mg/kg, with the first dose administered on August 3, 2010 (Table 2). The patient had no relapses during the study follow-up. Similar to Case 1, due to the premature termination of the ORACLE-MS study, the patient completed his end-of-trial visit on July 7, 2011.

**Figure 7:**
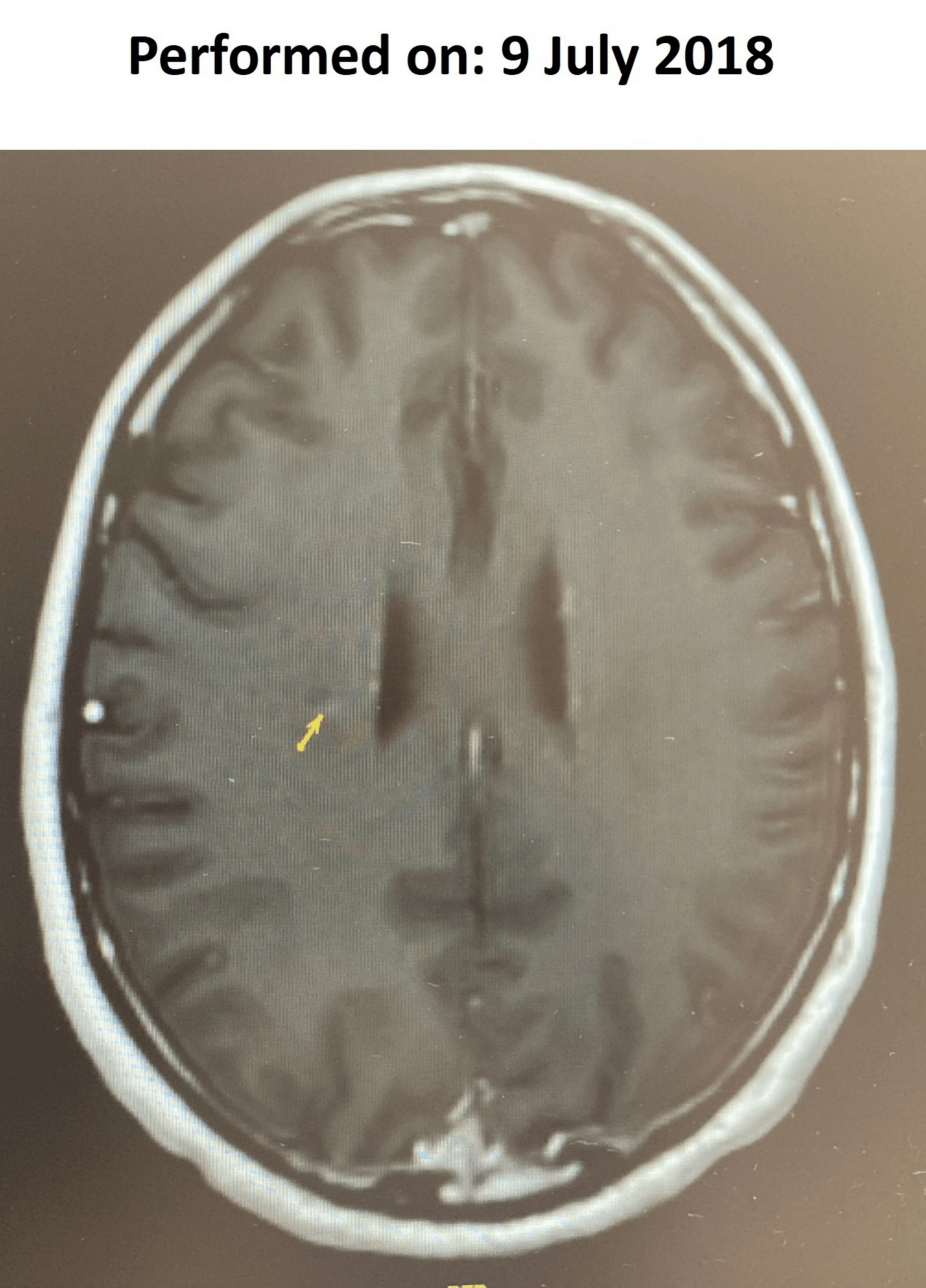
Subsequent cranial magnetic resonance imaging scan with contrast for Case 2

In January 2015, the patient experienced a second clinical event (non-enhancing injury; lower limb paresthesia, but without weakness), and based on an MRI scan, he was diagnosed with MS. He did not receive any treatment, and his symptoms had resolved after two weeks. A follow-up MRI in August 2017 showed no disease activity; however, in July 2018, he had one Gd+ right paraventricular lesion, and RMS was diagnosed (Figure 7). In October 2018, the patient started a second course of cladribine treatment. On August 27, 2020, the patient enrolled in the CLASSIC-MS study. In September 2020, his EDSS and EQ-5DL scores had increased slightly, but SDMT remained unchanged (Figure 6). In June 2021, his EDSS score had increased to 3.0 and remained at 3 until they were last measured in August 2023. However, his EQ-5DL and SDMT scores remained stable.

There were no new clinical events or disease progression following his second full course of cladribine in 2018. Follow-up MRI scans from September 2019 to November 2023 showed no signs of disease activity. The second course of cladribine was generally well tolerated. Although the patient’s lymphocyte count dropped below normal levels when he presented with grade 1 lymphopenia on January 31, 2020 (0.92 × 10^3^/μL) and March 3, 2020 (0.83 × 10^3^/μL) (Figure 2), the count subsequently recovered without any active management of lymphopenia. His lymphocyte count has remained normal as of August 2023.

## Discussion

The two cases described here involve patients who experienced their first relapse of MS as long as ~10 years (Case 1) and eight years (Case 2) after initial treatment for early MS with a single course of cladribine. Both were then retreated with a second course of cladribine for RMS, thus highlighting not only the long-term efficacy of the drug but also that retreatment is well tolerated.

An MS diagnosis can usually be made after a single clinical event, in conjunction with an MRI scan showing disease dissemination over time [6,8]. Early treatment initiation is essential, as initiation after the first clinical demyelinating event can delay the time until subsequent events or an MS diagnosis [6]. It is well-established that early treatment initiation allows for prolonged disease control and better treatment outcomes [7]. Both patients reported here had initially received a CIS diagnosis. Notably, the McDonald criteria for MS diagnosis were updated after the ORACLE-MS study was initiated [8]. Therefore, some of the patients enrolled in the study at the time would have met the current diagnostic criteria for MS [7]. These results support the early use of cladribine in patients with CIS, which improves treatment outcomes for patients with MS overall. This is in line with previous MS studies showing that DMTs in both treatment-naïve patients and those who have switched from one previous therapy have better treatment outcomes with cladribine than those who switched from two or more previous therapies [14].

In line with data from the CLASSIC-MS study [12], the two patients discussed in these case reports did not require further DMTs for at least 8-10 years after receiving an initial cladribine course (cumulative dose of 3.5 mg/kg), thereby confirming its long-lasting effects. In this sense, a recent expert consensus recommends that patients who had received two courses of cladribine (i.e., complete treatment) and were stable during years 3 and 4 may undergo annual MRI scans and, if available, neurofilament light chain measurement to monitor for disease reactivation [15]. If RMS occurs, an additional course of cladribine should be offered (i.e., a cumulative dose of 7 mg/kg) [13]. The consensus also underlined that in the instance where clinical or radiological activity is absent but the patient presents with poor prognostic factors, cladribine retreatment should be considered as a preventative measure against disease progression; in cases where disease activity is higher than that observed before cladribine initiation, switching to another treatment should be considered [15].

The favorable efficacy and safety profile of cladribine results from its unique physicochemical properties, which produce selective long-term depletion of proinflammatory memory B cells [16] and a modest reduction of regulatory B and T cells, thereby limiting disease progression by facilitating immune tolerance against self-pathogenic cells and suppressing self-mediated inflammation [17]. Historically, T cells were considered the key immune mediators underlying the pathogenesis of MS; however, B cells are now recognized as central mediators, following observation of their presence in peripheral blood and CNS compartments in patients with MS [2]. Such evidence highlights the potential role of B cells in antibody and cytokine production as well as antigen presentation [2]. Though cladribine also exerts modest effects on CD8 and plasma cells, their limited depletion allows for sustained and durable protection from infections. The innate immune system remains unaffected by cladribine [16]. The two-year MAGNIFY-MS study, in which patients with active RMS were treated with cladribine, showed that long-term reductions in proinflammatory B-cell cytokines (interleukin (IL)-6+) and T-cell cytokines (granulocyte-macrophage colony-stimulating factor, tumor necrosis factor-alpha, and interferon-gamma) paired with increases in anti-inflammatory T-cell cytokines (IL-4+) led to the re-establishment of immune tolerance required for long-term MS control. Additionally, novel results indicated increases in CD16low CD56bright and NKp46 cell populations [18].

Cladribine has an acceptable safety profile in adults with RMS [19]. The most frequently occurring cladribine-induced AE in this patient population is transient lymphopenia due to the selective depletion of lymphocytes [3]. In both cases reported here, Grade 1 lymphopenia occurred after the second course of cladribine but did not warrant treatment modification or discontinuation. Similarly, a post-hoc exploratory analysis of the CLARITY, CLARITY-extension, and ORACLE-MS studies found that CD19+ B-cell and CD4+/CD8+ T-cell counts decreased after cladribine 3.50 mg/kg treatment but had recovered to normal levels by the end of the study year [20]. These results further support the use of cladribine as an effective and generally safe long-term therapy for patients with RMS compared with other DMTs. Short dosing schedules and low monitoring requirements make cladribine an attractive therapeutic option for treatment-naïve patients and those who relapse (as well as those who are stable but experience AEs) on moderately effective DMTs [5]. Nevertheless, to reduce the risk of cladribine discontinuation due to AEs, patients should be encouraged to follow health guidelines, such as routine cancer screening [5].

## Conclusions

These case reports describe the treatment of MS that only relapsed 8-10 years after receiving one course of cladribine treatment. After the second course of cladribine, both patients remained clinically stable with no disease activity, although low-grade lymphopenia was observed. These cases provide real-world evidence of the long-term effectiveness and safety of cladribine in patients with early-stage MS and RMS and highlight the clinical value of offering cladribine in case of disease relapse.

The successful treatment of MS requires specialists to have a deep understanding of patient profiles, a readiness to utilize clinical and radiological tools for disease diagnosis and monitoring, and an in-depth knowledge of cladribine treatment and retreatment in early-stage MS and RMS.
